# Health-related quality of life in patients with low back pain in a low resource setting: a cross-sectional study at a tertiary hospital in Uganda

**DOI:** 10.4314/ahs.v23i1.59

**Published:** 2023-03

**Authors:** Robert Amesiya, Mallon Nyati, Gonzaga Waiswa, Erisa S Mwaka

**Affiliations:** 1 Department of Orthopaedics, College of Health Sciences, Makerere University, Kampala, Uganda; 2 Upper West Regional Hospital, Ghana Health Services, Wa, Ghana; 3 Department of Orthopaedics, Mulago National Referral Hospital, Kampala, Uganda; 4 Department of Anatomy, College of Health Sciences, Makerere University Kampala, Uganda

**Keywords:** Low back pain, Quality of life, Health-related

## Abstract

**Background:**

Low back pain is the leading global cause of years lost to disability. The study aimed to assess the health-related quality of life in patients with low back pain attending an outpatient clinic at a national referral hospital in Uganda

**Methods:**

This was a hospital based cross-sectional study that involved 250 adult patients with low back pain. Data were collected using the modified short form-36 Health Survey questionnaire. Data were summarised using descriptive statistics. Analysis of Variance, the F-test and linear regression analysis were used for inferential statistics.

**Result:**

Majority of participants were female (66.4%) with a mean age of 60 years (SD 12.9, range 20- 87) and 44.6% were manual labourers. 70% of participants had had low back pain for more than one year and 74% had neuropathic symptoms. The total quality of life of participants was poor with a mean score of 31.9 (SD 15.6). The factors that significantly influenced quality of life included performing manual work (p=0.01), being unemployed (p=0.027) and weakness in the lower limbs (p=0.01).

**Conclusion:**

Patients with low back pain had a poor quality of life that was significantly influenced by being unemployed, doing manual work and clinical features of nerve compression.

## Introduction

Low back pain (LBP) is relatively common and is increasingly recognized as a major health problem in Africa. The global age-standardized point global point prevalence of chronic LBP is estimated at 7.5% 1. A systematic review of 65 epidemiological studies across Africa reported a pooled adult LBP prevalence of 39% and an average lifetime prevalence of 47% 2. This pooled prevalence is higher than the 28.8% that was reported among adult Americans^3^. The lifetime prevalence of LBP is estimated to be as high as 84%, with chronic LBP estimated at 23%; and 11-12% of the population being developing disability due to LBP 4. Low back pain affects individuals of all ages 3 and is the leading global cause of years lost to disability 5, years lived with disability 1 and absenteeism from work 6, 7. Low back pain is so prevalent that it was ranked in the top 10 causes of years lived with disability in the 2016 Global Burden of Disease Study 8. In Uganda, the prevalence of LBP at a national referral hospital was reported at 20% 9.

Low back pain has considerable adverse effects on the health-related quality of life (QOL) of affected people. A patient with LBP not only suffers from physical discomfort, but also from functional limitation, which might cause disability and interfere with their QOL 12. There is a general agreement among researchers that QOL is a multidimensional concept comprising physical well-being, social well-being, and emotional well-being 10. Longitudinal studies have demonstrated association of back pain with functional disability and work incapacity, mental health problems, avoidance of certain activities, increased healthcare utilization and unemployment 11–14. Large-scale epidemiological studies show that recurrence is one of the major characteristics of LBP and often results in chronic disease 5. Chronic LBP has been associated with greater unemployment rates, pain-related limitation of function, seeking medical care and poorer self-related health 15, 16.

Factors associated with QOL in patients with LBP have been well-documented in Caucasian and Asian populations and are used to plan therapeutic interventions 10,11,12. However, this may not be true in sub-Saharan Africa including Uganda, because of the dearth of literature on LBP. Despite LBP being a fairly common disorder there have only been a few studies investigating the epidemiology of LBP in sub-Saharan Africa with a greater majority of them coming from Nigeria and South Africa 2,17. Further, there is limited literature on the QOL of patients with LBP in Africa. The Uganda National Clinical Guidelines 18 have a section on the management of back and bone pain that is very brief and does not provide adequate guidance on the management of back pain. There is thus need for research to generate valuable empirical evidence to influence policy change to modify the national treatment guidelines and improve guidance on the management of back pain. This study aimed to investigate the health related QOL of patients with LBP attending an out-patient clinic at a tertiary hospital in Uganda. In the context of LBP, understanding the predictors of QOL may help improve the clinical management of patients by extending the assessment process beyond the traditional, and clearly insufficient, clinical and functional disability variables 19, 20. Establishing the QOL and predictors of LBP may also help predict those at risk and thus accordingly guide prevention and treatment for LBP these settings.

## Methods

### Study Population and Design

This was a cross-sectional study conducted between April 2014 to May 2015, that investigated the QOL of patients with LBP attending an specialized spine outpatient's clinic at a national referral and teaching hospital for Makerere University. The hospital serves both referrals from peripheral hospitals and the general population in central Uganda. Two hundred fifty adult participants with non-traumatic LBP were recruited on their routine clinic visits using non-probability consecutive sampling. The sample size was calculated using the www.openepi.com online proportions sample size calculator for a 62% average lifetime prevalence of LBP among adults in Africa (17) and confidence limits of 5% for a power of 90.

### Assessment of QOL

Quality of life was assessed using the multi-dimensional and widely used Short Form-36 (SF-36) Health Survey tool (version 2.0) 21. The SF-36 questionnaire has 36 items that measure the health concepts of physical functioning, role limitations due to physical health problems, body pain, general health, vitality, social functioning, role limitations due to emotional problems, and mental health. It also contains a single item that examines change in health over time 21. The advantage of this questionnaire is that the SF-36 achieves the best balance between length, reliability, validity, responsiveness, and experience even in large populations of patients that complain of LBP/span> 21. Extensive psychometric testing of the SF-36 has been conducted in the United States 22–24 and other countries 25–29. It has also been validated in a few African countries 30–32.

To score the SF-36, scales are standardized with a scoring algorithm or by the SF-36 version 2 scoring software to yield two summary scores, the physical component summary (PCS) and the mental health component summary (MCS). The scores are converted to range from zero where the respondent has the worst possible health to 100 where the respondent is in the best possible health on the assumption that each question carries equal weight. The lower the score the more disability and the higher the score the less disability. A mean score of less than 50 indicates a health status that is below average 21.

We recoded the questionnaires basing on the scoring rules for SF-36 21, 33 and items in the same scale were averaged together to create 8 subscales. Summary measures of physical health, mental health and mean QOL score were computed. The tool was translated into Luganda, the most widely spoken language in central, mid-west and eastern Uganda. The questionnaire was administered in English and Luganda by the first author (RA) and two well trained research assistants after obtaining written informed consent. The tool was either self-administered or interviewer-assisted depending on the ability of the participants to read and write. Questionnaire completion on average took 30 minutes. Relevant clinical information about the respondents was obtained from their medical records.

### Data analysis

Association between QOL, the Mental Component Score and Physical Component Score was performed using Analysis of Variance (ANOVA) and the F-test. Variables with a p < 0.2 at univariate level were selected for multivariate analysis. Linear regression assumptions were assessed and dummy variable regression was employed to compute regression coefficients and 95% confidence interval. Goodness of fit of the model was assessed based on the adjusted sums of squares, and normality of the error terms. The level of significance was set at p < 0.05.

### Ethical considerations

The study was approved by Makerere University School of Medicine Research Ethics Committee. Written informed consent was obtained from all participants prior to enrolment in the study. All participants were assured of confidentiality. Participants received transport reimbursement and were compensated for their time.

## Results

A total of 250 patients with LBP participated in the study; a majority of which were female (66.4%). The mean age of participants was 60 years (SD 12.9, range 20- 87) and 44% were manual labourers. Participants' socio-demographic characteristics are summarized in Table 1.

**Table 1:** Demographic characteristics and clinical presentation

	Frequency (%), n= 250
**Gender**	
Male	84 (33.6)
Female	166 (66.4)
Mean QOL	
**Age**	
20–29	12 (4.8)
30–39	31 (12.4)
40–49	74 (29.6)
50–59	74 (29.6)
≥ 60	59 (23.6)
**Occupation**	
Office worker	41 (16.4)
Manual labourer	110 (44)
Market vendor	37 (14.8)
Unemployed	26 (10.4)
House wife	30 (12)
Student	6 (2.4)
**Clinical characteristics**	
**Duration of LBP**	
< 6 months	47 (18.8)
>6 months < 1 year	28 (11.2)
>1 year	175 (70)
**Numbness in lower limbs**	
Present	185 (74)
Absent	65 (26)
**Lower limb weakness**	
Present	143 (57.2)
Absent	107 (42.8)
**Paraesthesia in lower limbs**	
Present	181 (72.4)
Absent	69 (27.6)

A greater majority of participants (70%) had suffered from LBP for more than one year. Most participants reported having neuropathic symptoms as shown in Table 1.

The mean QOL was 31.9 (SD 15.6). Females had a relatively better quality of life than males but the difference was not statistically significantly (F= 0.32, p= 0.57). The age (p= 0.001) and occupation (p= 0.001) of the participants significantly affected the participants' QOL as shown in Table 2. The QOL decreased with increasing age, with the 50- 59-year age group having the lowest QOL scores. Students and office workers had better QOL scores than the other occupations.

**Table 2:** The association between quality of life and socio-demographic variables

Variable	Mean QOL ±std	F-test	P-value
**Gender**			
Male	31.2 ± 15.5	0.32	0.573
Female	32.4 ± 15.9		
**Age group**			
20–29	40.1 ± 19.3		
30–39	40.8 ± 20.1		
40-49	32.8 ± 16.6	4.82	0.001*
50–59	28.5 ± 12.9		
>=60	29.3 ± 12.4		
**Occupation**			
Office Worker	39.0 ± 22.1		
Manual Labourer	27.5 ± 11.3		
Market Vendor	34.4 ± 17.1		
Unemployed	27.9 ± 9.9	6.09	0.001*
Housewife	36.7 ± 15.3		
Student	46.7 ± 18.6		

* P< 0.05

The QOL generally decreased with increase in duration of LBP however, this association was not statistically significant (F=0.1, p= 0.67). Participants with numbness (p= 0.001), paraesthesia (p= 0.001) and weakness (p=0.001) in the lower limbs had significantly lower QOL (Table 3).

**Table 3:** Association between Quality of life and clinical variables

Variable	Mean±std	F-test	p-value
**Duration of LBP**			
Less than 6 months	33.7 ± 15.6	0.40	0.671
6 months to 1 year	32.7 ±15.3		
More than one year	31.5 ± 15.9		
**Lower limb numbness**			
Present	29.8 ± 14.1		
Absent	38.4 ± 18.5	15.44	0.001*
**Lower limb paraesthesia**			
Present	29.1 ± 13.4		
Absent	39.7 ± 18.6	25.06	0.001*
**Lower limb weakness**			
Present	27.3 ± 11.6	34.78	0.001*
Absent	38.4 ± 18.1		

* P< 0.05

When variables with p< 0.2 were analysed together in a multivariable model the following remained variables significant: being a manual labourer (p< 0.001), being unemployed (p= 0.027), and having weakness in lower limbs (p< 0.001). All the other variables became non-significant (Table 4).

**Table 4:** Regression analysis of predictors of QOL of LBP patients

QOL	Univariate analysis			Multivariate analysis		

	Coef (β)	P-value	95% CI	Coef (β)	P-value	95% CI
**Gender**						
Male	1					
Female	0.094	0.596	-0.257 – 0.446			
**Age group**						
20 – 29	1				1	
30 – 39	0.030	0.946	-0.839 – 0.899	0.398	0.924	-0.783 – 0.863
40 – 49	-0.589	0.146	-1.385 – 0.206	-0.326	0.408	-1.104 – 0.450
50 – 59	-0.960	0.018*	-1.756 – -0.164	-0.624	0.119	-1.411 – 0.161
≤ 60	-0.859	0.038*	-1.668 – -0.493	-0.365	0.370	-1.167 – 0.436
**Occupation**						
Office workers	1				1	
Manual labourer	-0.863	0.001*	-1.324 – -0.402	-0.651	0.001*	-1.112 – -0.189
Market vendor	-0.316	0.277	-0.887 – 0.255	-0.264	0.341	-0.810 – 0.281
Unemployed	-0.797	0.014*	-1.429 – -0.165	-0.728	0.027*	-1.375 – -0.081
Housewife	-0.054	0.860	-0.659 – 0.551	-0.203	0.490	-0.782 – 0.375
Student	0.709	0.206	-0.391 – 1.810	0.214	0.706	-0.782 – 0.375
**Duration of LBP**						
Less than 6 months	1					
Between 6 – 1 year	-0.836	0.793	-0.711 – 0.544			
More than 1 year	-0.222	0.314	-0.654 – 0.209			
**Numbness**	-0.713	0.001*	-1.081 – -0.344	0.171	0.497	-0.325 – 0.669
**Paraesthesia**	-0.882	0.001*	-1.238 – -0.527	-0.465	0.063	-0.955 – 0.025
**Lower limb weakness**	-0.936	0.001*	-1.251 – -0.621	-0.604	0.001*	-0.969 – - 0.238

* P< 0.05

The association between socio-demographic variables and clinical presentation on the Mental Component Summary Score (MCS) and Physical Component Summary Scores (PCS) of QOL are presented in Table 5. There was significant difference between the MCS and PCS among the different age groups (p=0.014) and occupations (p=0.001). The PCS and MCS decreased with increasing age. The unemployed and manual labourers had the lowest MCS and PCS. The presence of numbness (p<0.001), paraesthesia (p<0.001) and weakness (p<0.001) in the lower limbs significantly influenced the physical and mental well-being of participants.

**Table 5:** Association between the Physical component and mental component scores on demographic characteristics, occupation and clinical presentation

	Physical component score			Mental component score		
Variable	Mean±std	F-test	P-value	Mean±std	F-test	P-value
**Gender**						
Male	27.3±16.4	0.10	0.959	35.2±17.2	0.96	0.327
Female	27.4±17.5			37.4±17.3		
**Age group**						
20–29	35.1±21.9			45.1±20.3		
30–39	36.5±21.4			44.9±21.6		
40–49	286±169	4.59	0.014*	36.9±177	3.54	0.008*
50–59	23.4±14.4			33.6±15.4		
>=60	24.4±14.9			34.2±14.1		
**Occupation**						
Office Worker	35.5±23.8			42.6±22.9		
Manual Labourer	22.3±12.9			32.7±13.3		
Market Vendor	30.7±15.6	5.38	0.001*	38.1±20.6	5.08	0.002*
Unemployed	24.3±13.9			31.4±8.9		
Housewife	31.1±17.9			42.1±16.3		
Student	37.6±20.9			55.7±18.4		
**Duration of** **LBP**						
Less than 6 months	28.8±18.4			38.6±16.9		
6 months to 1 year	27.7±18.1			37.6±15.8		
More than one year	26.9±16.7	0.24	0.790	36.0±17.6	0.46	0.634
**Lower limb** **numbness**						
Present	25.3±15.5	10.72	<0.001*	34.2±15.4	15.09	<0.001*
Absent	33.3±20.2			43.6±20.2		
**Lower limb** **paraesthesia**						
Present	24.8±14.9	15.19	<0.001*	33.3 ±14.7	27.1	<0.001*
Absent	34.1±20.7			45.4±20.2		
**Lower limb** **weakness**						
Present	22.8±13.3	27.17	<0.001*	31.7±13.1	29.8	<0.001*
Absent	33.6±19.6			43.2±19.9		

## Discussion

Overall, the vast majority of participants had chronic pain with neurological symptoms. The overall QOL was poor and was significantly influenced by being a manual labourer, unemployed, and having weakness in the lower limbs.

The results of the study reported here do not differ from other studies which have reported the negative impact of LBP on QOL 34–38. The very low QOL reported in this study shows the extent to which LBP affects people's activities of daily living. A poor QOL may adversely affect a person's independence, productivity and may cause significant loss of time at work and lessens the individual's ability to compete on the job market. This may be one of the factors that may partly explain the lack of gainful employment among the majority of participants.

It is not surprising that unemployment and being a manual labourer were significant predictors of low QOL in this study. Uganda has an agriculture based economy with almost 70% of the population surviving on subsistence farming where people use traditional methods of farming, that majorly require manual labour 39. The agriculture sector is reported to have the highest relative risk for LBP and is an important cause of disability and poor quality of life 40. Relatedly, many people in Uganda work in the informal sector, which also involves a lot of manual work. Manual workers tend to perform heavy duties for long durations which may lead to early degenerative disease with resultant nerve compression 41, 42. Mechanical compression of the nerve roots in the lumbar spine by the nucleus pulposus and inflammatory granulation tissue results in radicular pain, numbness and paraesthesia to the lower limbs in LBP patients 43–45.

Unemployment was found to be a predictor of low QOL in our participants (p=0.01) and it significantly influenced the MCS (p=0.01). This finding is consistent with other studies that have reported significant association between unemployment and low MCS of QOL 46, 47. Unemployment seriously impacts an individual's ability to fend for his/her family and could have psychosocial implications^48^–^50^. Many unemployed individuals become depressed; the physiological aspects of such depression worsen the prognosis of LBP and unfortunately, its effect is underestimated and, poorly recognized and treated by clinicians^47^. Most clinicians concentrate more on obtaining an accurate diagnosis and alleviating the patient's symptoms with little emphasis on any psychosocial factors that may be aggravating symptoms. Depression, somatization, fear-avoidance beliefs, anxiety and stress have all been reported to be relatively common in LBP patients^51^–^53^. Personal beliefs, perceptions and expectations about pain, recovery and work have been reported to contribute to pain and disability especially in working populations. Therefore, management of patients with LBP should be multi-disciplinary and multi-faceted to ensure that both physical and psychosocial aspects are comprehensively investigated and addressed. Treatment to alleviate pain and improve function is as important as ensuring good psychological well-being.

Another factor that negatively affected QOL in this study was the presence of symptoms of nerve compression. This finding is consistent with other studies that have reported a considerably lower QOL and a higher degree of functional disability in patients with neuropathic pain compared with the general population 54–57. Participants with neuropathic symptoms had significantly lower PCS and MCS, and also perceived their general health as poor. In addition, patients with lower limb weakness had significantly worse QOL than those without (p=0.001); and this has also been reported by several studies 58, 59. Neuropathic symptoms lead to overall poor prognosis, and may be a predictor for chronicity and long-term disability^9^. Neuropathic symptoms may result in loss of independence thereby curtailing one's ability to carry out activities of daily living such as lifting, climbing stairs or walking and self-care 56. These symptoms may also disrupt people's family roles, relationships, destroyed their career and may contribute to depressive symptoms 34–38, 46.

Clinicians should therefore be cognizant of the profound impact that neuropathic pain can have on QOL, especially mental functioning. Maintaining independence, improving physical and emotional well-being are important goals that patients and clinicians can work towards together.

The study had a few limitations; our findings may not be generalizable because the study was conducted in a specialised spine clinic at a national referral hospital and non-probability consecutive sampling was used. However, they give a snapshot of the quality of life of patients with LBP in Uganda. In order to make generalizable conclusions, a population-based study using a probability sampling is recommended to survey the QOL of LBP patients in the country.

Cross-cultural content validity of the SF-36 questionnaire was assumed in this study. This presents a major limitation when comparing the concept of QOL in different cultures, as the respondents' understanding of the questions might vary. Bias was minimized by ensuring that the interviewers were well-trained in the administration of the questionnaire, and that it was translated into the local language with forward and backward translation.

## Conclusion

Evidence from this study shows that participants with LBP had poor health-related quality of life; and this was significantly affected by unemployment, engaging in manual work and having symptoms of nerve root compression. Management of patients with LBP should be multi-disciplinary and multi-faceted to ensure that both physical and psychosocial aspects are comprehensively investigated and addressed. There is need for more empirical research to explore potentially relevant psychosocial factors influencing health-seeking.
